# Neural correlates of linguistic collocations during continuous speech perception

**DOI:** 10.3389/fpsyg.2022.1076339

**Published:** 2022-12-23

**Authors:** Armine Garibyan, Achim Schilling, Claudia Boehm, Alexandra Zankl, Patrick Krauss

**Affiliations:** ^1^Chair of English Philology and Linguistics, University Erlangen-Nuremberg, Erlangen, Germany; ^2^Linguistics Lab, University Erlangen-Nuremberg, Erlangen, Germany; ^3^Neuroscience Lab, University Hospital Erlangen, Erlangen, Germany; ^4^Cognitive Computational Neuroscience Group, University Erlangen-Nuremberg, Erlangen, Germany; ^5^Pattern Recognition Lab, University Erlangen-Nuremberg, Erlangen, Germany

**Keywords:** collocations, electroencephalography, event related potentials, neurobiology of language, neurolinguistics, naturalistic continuous speech, cognitive computational neuroscience, natural language processing

## Abstract

Language is fundamentally predictable, both on a higher schematic level as well as low-level lexical items. Regarding predictability on a lexical level, collocations are frequent co-occurrences of words that are often characterized by high strength of association. So far, psycho- and neurolinguistic studies have mostly employed highly artificial experimental paradigms in the investigation of collocations by focusing on the processing of single words or isolated sentences. In contrast, here we analyze EEG brain responses recorded during stimulation with continuous speech, i.e., audio books. We find that the N400 response to collocations is significantly different from that of non-collocations, whereas the effect varies with respect to cortical region (anterior/posterior) and laterality (left/right). Our results are in line with studies using continuous speech, and they mostly contradict those using artificial paradigms and stimuli. To the best of our knowledge, this is the first neurolinguistic study on collocations using continuous speech stimulation.

## Introduction

How is natural language processed in the brain? Since decades this issue is tackled from different directions. On the one hand, in experimental neuroscience various neuroimaging techniques are applied to find neural correlates of speech perception in the brain (Schilling et al., 2021). On the other hand, computational linguistics tries to use computational models to unravel the mystery of language processing (Chowdhury, 2003; Nadkarni et al., 2011). While before the 1980s these computational approaches were mainly based on finding and applying strict syntactic rules, the field changed toward statistical natural language processing (Klein, 2005; Nadkarni et al., 2011). Thus, the recent advances in artificial intelligence research were a turning point in the field. Today computational linguistics also called natural language processing (NLP) is based on large text corpora which are used to train artificial neural networks (Klein, 2005). One field, which made significant progress in the last years and demonstrates the huge impact of this Big Data approach in combination with modern AI systems on natural language processing, is machine translation MT (Volkart et al., 2018; Rescigno et al., 2020; Yulianto and Supriatnaningsih, 2021).

However, we have not enough neural data recorded during naturalistic conditions like stimulation with continuous speech to apply the Big Data approach also in neurolinguistics. This is because so far, most neurolinguistic studies have mostly used experimental paradigms that are too simplified, e.g., by focusing on the processing of single words or isolated sentences. As a result, a large number of experimental variables known to affect natural language processing remains very poorly understood. Actually, “we currently cannot even be sure whether and how benchmark effects from traditional psycho-linguistic studies (e.g., word frequency and predictability effects on response times) generalize to more naturalistic situations.” (Hauk and Weiss, 2020). In contrast, the use of natural language, in particular connected speech, that resembles language as it is used in everyday life offers many advantages over well-controlled, simplified stimuli to study how language is represented and processed in the brain (Ding and Simon, 2012; Silbert et al., 2014; Brodbeck et al., 2018; Broderick et al., 2018; Deniz et al., 2019).

Although for some purposes it might be useful to think of language as a bag of words where the ordering of words does not matter, language is a highly structured system at multiple hierarchical levels where the presence of some linguistic structures can predict or determine the presence of others. Thus, language is fundamentally predictable. For instance, when encountering a ditransitive verb such as *give*, the language user expects the GIVER, the GIVEE and the THING GIVEN, because the argument structure construction implies these participants (Goldberg, 1995; Goldberg, 2006; Goldberg, 2019). In addition, for example, when encountering a new object for the first time, one would refer to it using the determiner upon the second encounter because of the definiteness marker.

Another way in which language can be predictable are collocations which are frequently co-occurring word combinations with a high strength of association, e.g.: *go home, annual meeting*, etc., being a ubiquitous phenomenon, collocations have received much attention from linguistic researchers. There are studies employing both paper-based (Herbst, 1996; Nesselhauf, 2005; Dąbrowska, 2014) and online/behavioral methods (Wolter and Gyllstad, 2013; Choi, 2017; Matsuno, 2017) to explore collocations. However, previous studies often looked at collocations in isolation. Among other ways, they would often administer paper-based multiple-choice tasks to reveal participants’ collocational competence or use the phrasal decision task to study the psycholinguistic validity of collocations. Yet, in real life, we do not encounter collocations in isolation. Therefore, this study has attempted to explore collocations using a method that does not rely on physical responses from participants and allows for the presentation of stimuli embedded in sentences and presented naturally. As far as relevant literature is concerned, there is just a handful of neurolinguistic studies of collocations, let alone ERP (event related potentials) studies. In particular, two of these studies are worth mentioning in this respect. Molinaro and Carreiras looked at figurative as well as literal interpretations of Spanish collocations (Molinaro and Carreiras, 2010). Using a Rapid Serial Visualization Task (RSVT), in which participants see sentences presented word-by-word in the center of the screen separated by a pre-defined inter-stimulus interval, they established that collocations in the figurative reading were associated with larger negativities in the N400^1^ time window in comparison with their literal readings suggesting that more processing load is required to integrate the distant meanings in figurative collocations. However, while the title of the paper contains the word ‘collocations’, what the authors mean and explicitly explain in the paper is a broad heterogeneous class of multi-word units, e.g.: collocations, idioms, clichés, proverbs, etc. Thus, the operationalization of collocations in their study is quite different from the strictly linguistic definition of collocations found in the traditional literature on collocations (John Rupert Firth, 1956; Hausmann, 1984; Sinclair, 1991).

The second study by Hughes (2018) comes the closest to our operationalization of collocations in that the difference between collocations and non-collocations is seen as purely quantitative rather than qualitative. So, she uses transitional probability (TP) of 0.01 to distinguish between the two conditions. In a series of experiments, and using the same methodology as Molinaro and Carreiras (2010), i.e., RSVT, Hughes (2018) claims that non-collocational bigrams are associated with a larger N400 in comparison with collocational bigrams since non-collocations are less expected than collocations, and the effect was right-lateralized. Yet, Hughes (2018) has only 15 collocational bigrams which she repeats twice to reach a sufficient number of trials, which is problematic since repetition of the same stimuli can lead to the reduction of the N400 amplitudes (Besson and Kutas, 1993). In general, a review of these studies leads to the two following concerns. While it can be argued that RSVT is a more natural task than, for example, a phrasal decision task, the question is whether the task could become even more ecological. In other words, the speed with which speakers experience language in real life is not pre-defined. Therefore, previous findings might have been influenced by this artificial character of the experimental design. In contrast, it is expected that measuring neural responses to collocations in naturalistic settings, e.g., during continuous auditory speech comprehension, will reflect the nature of collocation processing in a more realistic way. Next, the way collocations are operationalized in these studies calls first, for a more linguistic definition of collocation, and second, for a more realistic cut-off point between collocations and non-collocations as far as statistical measures of collocation strength are concerned.

We will conclude these section by presenting our expectations. Thus, given that the N400 is a marker of ease of cognitive processing, with more unpredictable and surprising items showing a larger N400, it was expected that non-collocations will be associated with larger negativities in the N400 time window. As far as the topography is concerned, we did not have any clear expectations because of the mixed findings in the literature. In particular, Hughes (2018) reports various distributions in a series of experiments (ranging from anterior through central to posterior scalp distributions). However, as far as lateralization is concerned, we expect larger effects in the right hemisphere. There are at least two reasons to suggest that. First, in one of the experiments done by Hughes (2018), a right-lateralized N400 was reported. Second, given that according to Van Lancker and Kempler (1987), familiar phrases are processed in the right hemisphere whereas novel ones are processed in the left hemisphere, we hypothesized that collocations (being familiar phrases) will be processed in the right hemisphere, and non-collocations (being novel language) in the left hemisphere.

## Materials and Methods

### Human participants

Participants were 31 (13 females, 18 males) healthy right-handed (augmented laterality index: *μ* = 83.8, *σ* = 20.8) and monolingual native speakers of German aged 20–68 years (*μ* = 27.4 years, *σ* = 9.0 years, < 30 years: *n* = 26, 30-39 years: *n* = 2, 40-49 years: *n* = 2, > 50 years: *n* = 1). They had normal hearing and did not report any history of neurological illness or drug abuse. They were paid for their participation after signing an informed consent form. Ethical permission for the study was granted by the ethics board of the University Hospital Erlangen (registration no. 161–18 B). For the questionnaire based assessment and analysis of handedness we used the Edinburgh Inventory (Oldfield, 1971). In order to avoid any unwanted familiarity or repetition effect, we excluded participants from our study that already read the novel or listened to the audio book (see below).

### Speech stimuli and natural language text data

As natural language text data, we used the German novel *Gut gegen Nordwind* (engl: Good against north wind) by Daniel Glattauer (© *Deuticke im Paul Zsolnay Verlag, Wien 2006*) which was published by *Deuticke Verlag*. As speech stimuli, we used the corresponding audio book which was published by *Hörbuch Hamburg*. Both the novel and the audio book are available in stores, and the respective publishers gave us permission to use them for the present and future scientific studies. This novel was chosen because it is written in contemporary, everyday-language and does not contain sexual, violence glorifying or otherwise offensive content. Book and audio book consist of a total number of 40,460 tokens (number of words) and 6,117 types (number of unique words). The total duration of the audio book is approximately 4.5 h. For our study, we only used the first 40 min of the audio book, divided into 10 parts of approximately 4 min (*μ* = 245 s, *σ* = 39 s). This corresponds to approximately 6,000 words, or 800 sentences, respectively of spoken language, where each sentence consists on average of 7.5 words and has a mean duration of 3 s. In order to avoid cutting the text in the middle of a sentence or even in the middle of a word, we manually cut at paragraph boundaries, which resulted in more meaningful interruptions of the text.

### Stimulation protocol

The continuous speech from the audio book was presented in 10 subsequent parts (*cf.* above) at a sensory level of approximately 30–60 dB SPL. The actual loudness varied from participant to participant. It was chosen individually to ensure good intelligibility during the entire measurement, but also to prevent it from being unpleasant. Simultaneously with auditory stimulation, a fixation cross at the center of the screen was presented all the time to minimize artifacts from eye movements. After each audio book part, three multiple-choice questions on the content of the previously presented part were presented on the screen in order to test the participants’ attention. Participants had to answer the questions by pressing previously defined keys on a keyboard. The total duration of the protocol is approximately 1 h.

### Generation of trigger pulses with forced alignment

In order to automatically create trigger pulses for both, the synchronization of the speech stream with the EEG recordings, and to mark the boundaries of words for further segmentation of the continuous data streams, forced alignment (Moreno et al., 1998; Yuan and Liberman, 2009; Katsamanis et al., 2011) was applied to the text and recording. For this study we used the free web service WebMAUS (Schiel, 1999; Kisler et al., 2017). It takes a wave file containing the speech signal, and a corresponding text file as input and gives three files as output: the time tags of word boundaries, a phonetic transcription of the text file, and the time tags of phone boundaries. Even though forced alignment is a fast and reliable method for the automatic phonetic transcription of continuous speech, we carried out random manual inspections in order to ensure that the method actually worked correctly. Although forced alignment is not 100% reliable, manual spot checks found no errors in our alignment, with an average temporal error below 10 ms. Of course, the high-quality recording of an audio book is among the best possible inputs for such software. For simplicity, we only used the time tags of word boundaries in this study.

### Speech presentation and synchronization with EEG

The speech signal was presented using a custom made setup. It consists of a stimulation computer connected to an external USB sound device (Asus Xonar MKII, 7.1 channels) providing five analog outputs. The first and second analog outputs are connected to an audio amplifier (AIWA, XA-003), where the first output is connected in parallel to an analog input channel of the EEG data logger in order to enable an exact alignment of the presented stimuli and the recorded EEG signals. In addition, the third analog output of the sound device is used to feed the trigger pulses derived from forced alignment into the EEG recording system *via* another analog input channel. By calculating the cross-correlation between the original and the recorder trigger pulses, an exact synchronization can be achieved.^2^ In doing so, our setup prevents temporal jittering of the presented signal caused by multi-threading of the stimulation PC’s operating system, for instance. The speech sound was presented open field via loudspeakers.

The stimulation software is implemented using the programming language *Python 3.6*, together with Python’s sound device library, the *PsychoPy* library (Peirce, 2007; Peirce, 2009) for the stimulation protocol, and the *NumPy* library (Harris et al., 2020) for basic mathematical and numerical operations.

### Electroencephalography and data processing

For EEG recordings we used the actiChamp amplifier from Brain Vision (Brain Products, Brain Vision, Morrisville, United States). The setup has 64 active electrodes, which were recorded with a sampling rate of 2.5 kHz and no further spectral filters, as filtering was performed after the measurement offline, during the evaluation procedure. Electrode impedance was tuned by the application of electrically conductive gel, so that the skin resistance at each electrode location was below 20 kΩ.

Further processing was performed using the Python library *MNE* (Gramfort et al., 2013, 2014). The data was band-passed filtered off-line at 0.1-30 Hz. For artifact rejection and instead of baseline correction (Alday, 2019), data were corrected using independent component analysis (ICA) and subsequently removing the first two independent components. Then, the data was epoched from 200 ms prior stimulus onset to 800 ms post stimulus onset. No baseline correction was applied since in the context of natural speech processing, period before stimulus onset was not period of inactivity.

Finally, evoked data for each participant followed by grand averages across 31 participants were created. The subsequent analysis of brain responses was planned in the following areas: left-anterior area (FC1, FC3, FC5, F1, F3, F5, F7, AF3, AF7), right-anterior area (FC2, FC4, FC6, F2, F4, F6, F8, AF4, AF8), left-posterior (CP1, CP3, CP5, P1, P3, P5, P7) and right-posterior (CP2, CP4, CP6, P2, P4, P6, P8).

For this study, we restricted our analyzes to sensor space, and did not perform source localization.

### Alignment and segmentation

Since we have both, the original audio book wave file together with the time tags of word boundaries from forced alignment, and the corresponding recordings of two analog auxiliary channels of the EEG, all 64 EEG recording channels could easily be aligned offline with the speech stream. Subsequently, the continuous multi-channel EEG recordings were segmented using the time tags as boundaries.

### Collocations

We restricted our study to adjective-noun bigrams, and identified 87 of such bigrams in the book. Subsequently, we checked their frequencies in the deTenTen13 part of the SketchEngine corpus which contains over 20 billion words. In addition, the MI values were extracted from the corpus. We only selected collocations with a high strength of association. All non-collocations had an MI lower than 4.83 and all collocations had an MI higher than 6.72. Applying these conditions resulted in 13 collocational adjective-noun bigrams. Finally, the number of non-collocations was chosen to match the number of collocations. Furthermore, the conditions were matched for frequency of individual words and word length. Hence, the only difference between the two conditions was that collocations were more predictable based on the higher MI value than non-collocations. Given that the noun was the critical word to which the brain response was time-locked, and that high-frequency words are associated with reduced N400 effects in comparison with low-frequency words (Barber et al., 2004), the bigrams were controlled for length and individual word frequency, not phrasal frequency. Operationalization of collcoations is based on a statistical measure of strength of association - mutual information (MI). MI was extracted from the SketchEngine corpus. There it is calculated as MI = log((AB * *N*)/(*A* * *B* * *K*))/log(2), “with A the frequency of the node word” (e.g., “Raum”), *B* the frequency of the collocate (e.g., “Luftleerem”), AB the frequency of collocate near the node word (e.g., “Luftleerem Raum”), *N* the total number of words in the corpus, and *K* the span of words (e.g., 3 to the left and 3 to the right of the node word). Using this formula and the frequency information from the deTenTen13 data base, we cross validated the MI values provided by SketchEngine. An MI of 5 was taken as a cut-off point between collocations and non-collocations with a buffer zone of approximately 2 units between the conditions. More information about the characteristics of collocations as well as non-collocations can be found in Table 1.^3^

**Table 1:** Item characteristics for collocations and non-collocations (translations into English in square brackets).

Word combination	Relative frequency (first word)	Relative frequency (second word)	Relative frequency (word combination)	Mutual information
Collocations				
Luftleerem Raum [empty space]	0.403	179.922	0.313	12,07
Zugekniffenen Augen [closed eyes]	0.052	186.364	0.039	11.97
Mulmiges Gefühl [queasy feeling]	1.581	123.234	0.701	11.81
Spitze Zunge [sharp tongue]	8.703	11.540	0.108	10.07
Niedrigem Blutdruck [low blood pressure]	63.948	6.629	0.425	9.97
Beruflichen Tätigkeit [professional activity]	57.828	67.397	2.249	9.17
Gestellten Fragen [asked questions]	16.690	468.665	3.116	8.64
Verheirateten Frauen [married women]	3.728	484.008	0.424	7.88
Dunkle Haare [dark hair]	61.371	57.298	0.709	7.66
Allerletzte Chance [very last chance]	2.483	148.129	0.067	7.50
Harmonische Beziehung [harmonious relationship]	15.426	85.785	0.181	7.10
Positiven Denkens [positive thinking]	144.827	31.704	0.607	7.05
Letztes Mal [last time]	548.940	209.305	12.105	6.72
Non-collocations				
Dauerhafte Liebe [abiding love]	37.418	158.959	0.018	1.63
Herrliches Gefühl [wonderful feeling]	43.970	123.234	0.132	4.61
Erhaltenen Sätze [received sentences]	0.008	101.133	0	0
Imposante Geste [impressive gesture]	8.223	8.452	0.001	3
Stilvoller Verlierer [stylish loser]	8.677	6.194	0	0
Realen Umgebungen [real environment]	36.225	73.968	0.076	4.83
Allfälligen Treffen [possible meeting]	3.824	39.096	0.001	0
Quirlige Person [lively person]	2.327	248.850	0.002	0
Markanten Anlass [distinctive reason]	9.582	52.683	0	0
Lustlosesten Antwort [dullest answer]	1.269	141.288	0	0
Schöne Homepage [nice homepage]	491.246	53.832	0.177	2.74
Ersehnte Meldung [long-desired message]	4.753	32.984	0.001	2.89
Zielstrebige Art [determined manner]	2.770	258.706	0	0

Note that all word combinations - except “Spitze Zunge” - are semantically transparent.

### Statistical analysis

For statistical analysis, permutation tests were computed for the latency window of 300–500 ms. (Maris and Oostenveld, 2007). The permutation tests were done in *RStudio* (RStudio Team, 2016) using the *Coin* package (Hothorn et al., 2008). The following setting was used for permutation testing (function “independence test”): asymptotic distribution, standardized scalar test statistic, two-sided alternative hypothesis.

## Results

**Figure 1:**
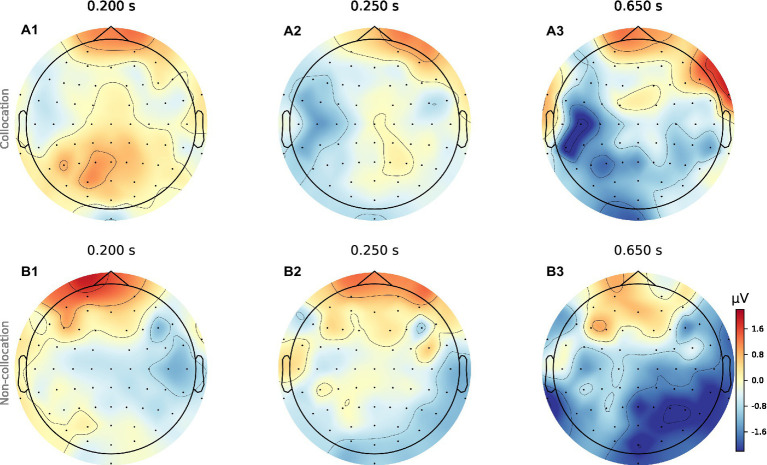
Topomaps of grand averages for collocations and non-collocations. The surface plots show the averaged neural signal (neural activity) of the cerebral cortex averaged over 31 participants for collocations (a1-a3) and non-collocations (b1-b3) stimulation at three different time points (latencies: 200 ms, 250 ms, 650 ms). Collocation (blue) vs. non-collocation (red).

**Figure 2:**
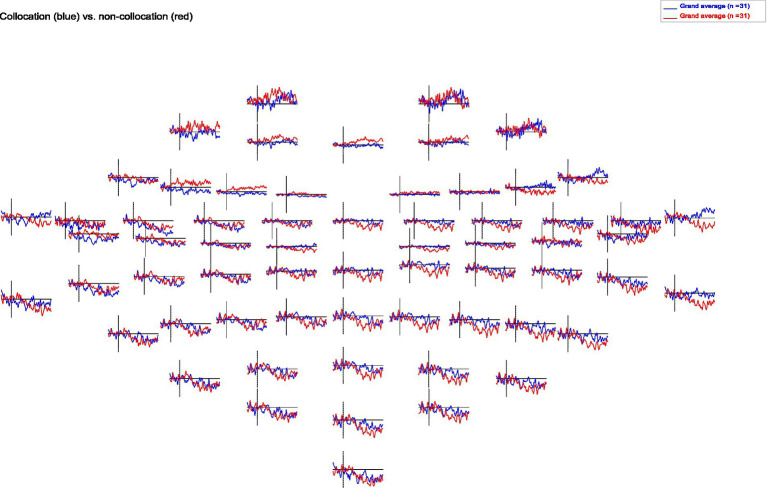
Topomap of grand averages of ERPs for collocations and non-collocations. Collocations (blue) cause larger N400 amplitudes in anterior brain regions symmetrically in both hemispheres compared to non-collocations (red). Additionally, collocations induce a clear negativity in the lateral posterior regions of the left hemisphere. In contrast, non-collocations cause an increased N400 amplitude in the right hemisphere.

When analyzing the resulting ERPs (Strandburg et al., 1993), we find that presentation of collocations compared to non-collocations causes larger N400 negativities in anterior brain regions symmetrically in both hemispheres. Additionally, collocations induce a clear negativity in the lateral posterior regions of the left hemisphere (Wernicke’s area), which starts at a latency of 250 ms and is most pronounced around 650 ms. However in contrast to that, in the right hemisphere non-collocations cause an increased N400 amplitude compared to collocations (see Figures 1, 2). This indicates that higher level linguistic structures are processed differently in the two hemispheres.

**Figure 3:**
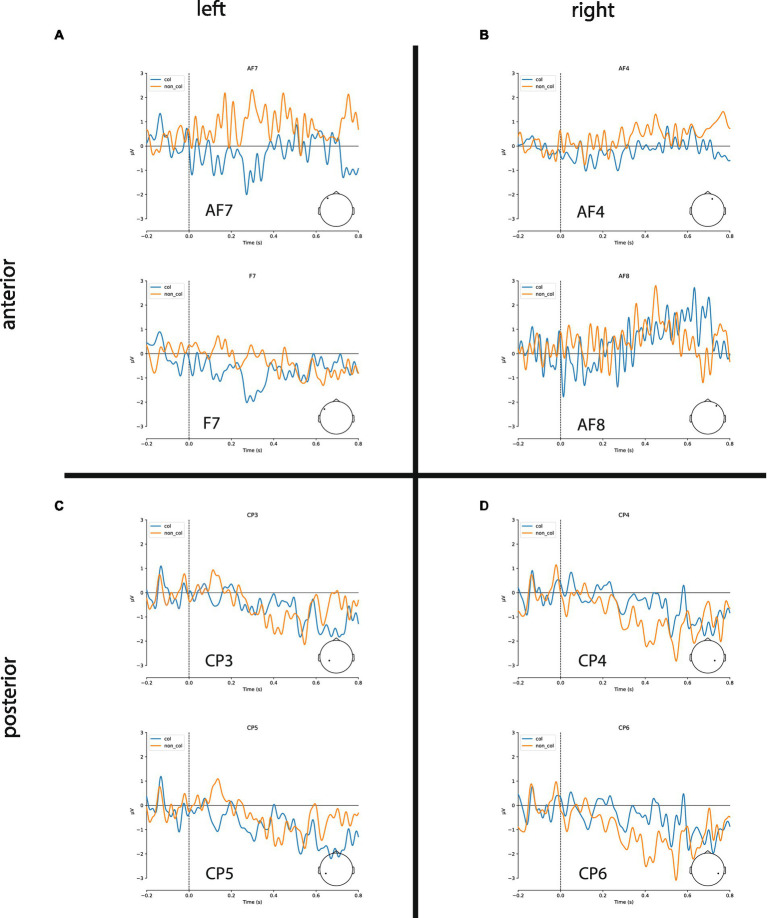
Grand averages of ERPs for exemplary channels. **(A,B)** Collocations (blue) cause larger N400 amplitudes in anterior brain regions compared to non-collocations (orange) in both hemispheres. **(C)** Collocations induce a clear negativity in the lateral posterior regions of the left hemisphere (Wernicke’s area). **(D)** Non-collocations cause an increased N400 amplitude in posterior regions of the right hemisphere.

In the following, we show the statistical analysis of the negativities within the time-window 300–500 ms after stimulus onset recorded at the four electrode sites (left/right anterior/posterior, for grand averages see Figure 3). The statistical analysis reveals that there are indeed larger negativities for collocations in the anterior area (left: −0.55 μV, right: −0.32 μV) compared to non-collocations (left: 0.31 μV; right: −0.08 μV). In the posterior area, the picture is mixed: similarly to the anterior area, in the left-posterior area collocations are marked by larger negativities (−0.21 μV) in comparison with non-collocations (−0.02 μV) whereas in the right-posterior area, the situation is opposite (collocations: −0.15 μV; non-collocations:−0.59 μV). We restricted our analysis to this time-interval, because we expect the N400 negativity there. This negativity is a marker of unpredictability and surprisal, and therefore a marker of higher processing load.

According to the results of the permutation tests in the time-interval of 300–500 ms, the observed amplitude differences between collocations and non-collocations were statistically significant at all four electrode sites (see Table 2), i.e., left/right anterior and posterior (*p* < 0.001 in each case).

**Table 2:** Mean amplitudes for collocations and non-collocations in the left/right anterior, posterior areas.

	LA	RA	LP	RP
Collocation	−0.55	−0.32	−0.21	−0.15
Non-collocation	0.31	−0.08	−0.02	−0.59
*p*-values	<0.001	<0.001	<0.001	<0.001

Finally, the procedure was repeated to find out whether there was any difference between collocation-processing at the four different recording sites. We show that collocation processing is highly lateralized with the most prominent effect at the anterior regions (*p* < 0.001).

## Discussion

Given that the N400 is a marker of ease of semantic processing with more unpredictable items showing a (larger) N400, we expected that non-collocations will be associated with larger negativities since they are more unpredictable than collocations. However, in fact, in the N400 time interval, collocations are associated with larger negativities in the anterior area as well as the left-posterior area compared to non-collocations (see Figure 1). This finding contradicts the results obtained by Hughes (2018) who found a larger N400 wave with anterior scalp distribution for non-collocational bigrams. That our findings are opposite to Hughes (2018) who operationalizes collocations in a similar way to ours is controversial. However, the non-collocational bigrams in Hughes (2018) are artificially created adjective + noun bigrams, although semantically plausible, that do not appear in the *British National Corpus* (BNC) and were presented in isolated sentences not united by a common context. In the context of the present study, non-collocations appeared in an audio book fragment which is a coherent piece of discourse. Therefore, it would be valid to assume that the non-collocational bigrams in Hughes (2018) were more unexpected than the non-collocations in the present study appearing in a natural piece of discourse. However, we also found that collocations were modulated by reduced negative activity in comparison with non-collocations in the N400 time window in the left-posterior area, which is in line with Hughes (2018) who found more negativity for non-collocations in all electrode sites tested, including the left-posterior area.

Our results can also be supported by Molinaro and Carreiras (2010) who studied the effect of unpredictability of complex prepositions in Spanish ending with either a predictable or unpredictable word, e.g.: *in support for/of*. Similarly to us, they found larger negativities (N400-700) to the predictable endings in comparison to the unpredictable ones. Thus, our findings in the anterior as well as left-posterior areas are in line with Molinaro and Carreiras (2010) since more predictable units exhibited a larger N400. A word of caution needs to be mentioned, though, due to the fact that unlike the present study, the critical (last) word in Molinaro and Carreiras (2010) was a preposition, that is a closed class and a function word. In our study, the critical word was a noun, i.e., an open class and a content word. We are not arguing that this should have necessarily impacted our results in terms of the predictability effects. However, a previous study by Schilling et al. (2021) reports a fundamental difference between the processing of content words versus function words in the brain, this fact as well as its possible relation to the interpretation of our results should not be left unnoticed.

Whereas previous literature supporting our findings comes from studies on visual language comprehension, the results from Koskinen et al. (2020) are especially relevant in the context of the present study since they were also obtained in the context of auditory speech processing. In this work, MEG brain activity was examined during continuous speech processing when participants listened to a 1-h audio book. The authors found effects of word predictability based on the contextual information in the left hemisphere that mainly involved temporal and frontal brain areas, which overlaps with the left anterior region defined in our study where the largest difference between conditions is observed. This finding can be used to argue that collocations as relatively predictable word combinations are predominantly associated with left anterior processing.

Finally, the study that comes the closest to our overall findings is one by Sereno et al. (2020) who looked at the effects of contextual predictability in reading and found widespread predictability effects in the N400 time window. In addition, the differences were most marked in the left anterior area with more negative amplitudes for high predictability words and more negative-going amplitudes for low predictability words in midline-central and midline-posterior electrode sites. As mentioned earlier, this perfectly matches our results where more predictable items (i.e., collocations) showed more negative-going amplitudes in the left anterior area, and more unpredictable items (i.e., non-collocations) showed negative-going amplitudes in the right-posterior area (see Figure 3).

As far as the laterality of the N400 is concerned, based on the results of the permutation tests, the effect of collocation was significant across all four brain areas. This partially confirms the findings of Van Lancker and Kempler (1987) who claimed that familiar phrases are processed in the right hemisphere whereas novel language is processed in the left hemisphere because the second largest difference between collocations and non-collocations is in the right-posterior area. However, as mentioned earlier, we found a statistically significant and the largest difference between collocations and non-collocations in the left-anterior area, which seems to contradict their findings. We will use our findings to argue that collocations, as defined in the context of the present study, do not share many features with the formulaic language described in Van Lancker and Kempler (1987) who although do not provide a list of the experimental items, still give a few examples of formulaic language used in the experiment, e.g.: *He’s turning over a new leaf*; *While the cat is away, the mice will play*. As visible from these items, these are examples of idioms which are both syntactically and semantically fixed, often representing one unit of meaning and therefore having strong imagery. Yet, collocations in our study are regular word combinations that are both syntactically and semantically transparent. That is why it can be argued that the presence of a large effect of collocation in the left-anterior area can be explained by the fact that collocations share some features with novel language in that they are analyzable multi-word units, whereas the presence of the collocation effect in the right-posterior area can be explained by its idiomaticity.

In addition, it is necessary to point out the importance of modality of the task. Holcomb and Neville (1991) studied semantic relatedness in connection with either visual or auditory modality. What they found was larger negativities in the N400 time window in the right hemisphere in the auditory task than in the visual task suggesting that the right hemisphere is responsible for processing prosodic cues in natural speech. Given that the present study is also based on brain responses to natural speech signal, our findings can be argued to be in line with those of Holcomb and Neville (1991) because the second largest difference between the two conditions was observed in the right hemisphere.

To sum up, our results show that collocations are a psychologically valid phenomenon by the presence of statistically significant effects in all four electrode sites tested. However, the exact configuration of this effect, e.g., amplitudes, lateralization, remains debatable. We argue that predictability as shown by collocations is modulated by larger negativities in the left anterior area in comparison with non-collocations, but smaller negativities in the right-posterior area. Hence, although we managed to find previous studies that support our findings, it needs to be stated that relating our results to previous literature is challenging because of the many various changes in the configurations of those studies,

- item selection criteria, modality of stimuli presentation, task, etc. Kutas and Dale (1997) say that ‘N400s do differ in latency and scalp distribution, even within presumably similar experimental tasks’ (p. 222). Thus, small changes of the configurations of a study, lead to different results. Yet, this work has contributed to studies on collocations in many ways. First, to the best of our knowledge, this is the first neurolinguistic study of collocations, let alone in the context of natural speech processing. Also, given that collocations are a ubiquitous phenomenon that we encounter daily in all kinds of discourse, we hope that this study will serve as starting point for more naturalistic studies of collocations, which will lead us closer to the understanding of how these multi-word units are processed in the brain.
  However, this study is just a pilot study, and further analyzes and experiments are needed to gain a more solid data base. Furthermore, the study provides evidence that the results of the neurolinguistic studies have to be accompanied by computer simulations, helping to generate hypotheses, which can be tested in the experiments. A lack of these hypotheses makes it nearly impossible to interpret the data. The computational approach can be combined with innovative evaluation techniques based on AI, e.g., dimensionality reduction techniques to account for neural activity spread over the complete cortex (Krauss et al., 2018a,b, 2021). This highly interdisciplinary approach based on modern evaluation techniques combined with computer models and strict hypotheses-driven research could potentially solve the problem of low reproducibility between different neurolinguistic and psychological studies (Maizey and Tzavella, 2019; Hensel, 2020).
  Furthermore, neuroscience can profit from recent advances in computational linguistics. In particular, deep artificial neural networks trained on language processing can serve as models for brain function, as argued by Kriegeskorte and Douglas (2018) and Krauss and Schilling (2020), who call that approach “Cognitive Computational Neuroscience” (CCN). In particular, artificial neural networks trained on extensive text corpora can be analyzed to generate hypotheses about important structures and processes involved in language processing. These hypotheses may be tested using neuroimaging data in order to find parallels between artificial and biological neural networks [*cf.* (Jonas and Kording, 2017)].
  Since contemporary AI systems largely lack biological plausibility, existing neural network models have to be made biologically more plausible by, e.g., generating hybrid models from standard machine learning and biologically inspired neuron models (Schilling et al., 2020a; Gerum and Schilling, 2021; Maier et al., 2022; Stoewer et al., 2022), applying biologically plausible learning rules (Gerum et al., 2020), or biological processing principles such as stochastic resonance and neural noise to make network models more stable (Krauss et al., 2016, 2018c; Schilling et al., 2020a,b; Yang et al., 2021).
  We conclude that, the approach to merge computational linguistics and neurolinguistics is not exclusively useful for neuroscience, but can also be a source of inspiration for novel and more efficient AI approaches (Hassabis et al., 2017).

## Data availability statement

The raw data supporting the conclusions of this article will be made available by the authors upon reasonable request, and to the extent consistent with privacy policies.

## Ethics statement

The studies involving human participants were reviewed and approved by Ethics board of the University Hospital Erlangen. The patients/participants provided their written informed consent to participate in this study.

## Author contributions

PK, AS, and AG designed the study. PK and AS supervised the study. AG, AS, and PK analyzed the data and wrote the manuscript. CB, AZ, AS, and PK performed the experiments. All authors contributed to the article and approved the submitted version.

## Funding

This work was funded by the Deutsche Forschungsgemeinschaft (DFG, German Research Foundation): grant KR 5148/2–1 (project number 436456810) to PK, and grant SCHI 1482/3–1 (project number 451810794) to AS. Furthermore, this work was funded by the Emerging Talents Initiative (ETI) of the University Erlangen-Nuremberg (grant 2019/2-Phil-01 to PK), and the Interdisciplinary Center for Clinical Research (IZKF) at the University Hospital of the University Erlangen-Nuremberg (grant ELAN-17-12-27-1-Schilling to AS).

## Conflict of interest

The authors declare that the research was conducted in the absence of any commercial or financial relationships that could be construed as a potential conflict of interest.

## Publisher’s note

All claims expressed in this article are solely those of the authors and do not necessarily represent those of their affiliated organizations, or those of the publisher, the editors and the reviewers. Any product that may be evaluated in this article, or claim that may be made by its manufacturer, is not guaranteed or endorsed by the publisher.

## Footnotes

^1^The N400 is a component of event-related potentials (ERP). It is a negative-going deflection that peaks about 400 milliseconds after stimulus onset, although it can extend over 250–500 ms. The N400 is part of the brain’s normal response to words.

^2^Note that, as an alternative approach for synchronization, the lab streaming layer (LSL) distribution (https://github.com/sccn/labstreaminglayer) which is a system for the unified collection of measurement time series in research experiments, could be used.

^3^Using the *SketchEngine*, word combinations were extracted from the German corpus deTenTen13 which at the time of analysis (June 2021) contained 19.8 billion words. Relative frequencies in Table 1 refer to frequency per million words.
